# International Neurotrauma Training Based on North-South Collaborations: Results of an Inter-institutional Program in the Era of Global Neurosurgery

**DOI:** 10.3389/fsurg.2021.633774

**Published:** 2021-07-29

**Authors:** Andrés M. Rubiano, Dylan P. Griswold, P. David Adelson, Raul A. Echeverri, Ahsan A. Khan, Santiago Morales, Diana M. Sánchez, Robson Amorim, Alvaro R. Soto, Wellingson Paiva, Jorge Paranhos, José N. Carreño, Ruy Monteiro, Angelos Kolias, Peter J. Hutchinson

**Affiliations:** ^1^INUB-Meditech Research Group, Neuroscience Institute, Universidad El Bosque, Bogota, Colombia; ^2^Meditech Foundation, Valle-Salud IPS Clinical Network, Cali, Colombia; ^3^Division of Neurosurgery, National Institute of Health Research (NIHR) Global Health Research Group in Neurotrauma, University of Cambridge, Cambridge, United Kingdom; ^4^Meditech Foundation, Neurotrauma and Global Surgery Fellowship Program, Cali, Colombia; ^5^Barrow Neurological Institute, Phoenix Children's Hospital, Phoenix, AZ, United States; ^6^Neurological Surgery Service, Aga Khan University, Karachi, Pakistan; ^7^Neurosurgery Training Program, Universidad de Ciencias Médicas, Havana, Cuba; ^8^Neurosurgery Program, Federal University of Amazonas, Manaus, Brazil; ^9^Neurosurgery Service, UROS Clinic, Neiva, Colombia; ^10^Neurosurgery Service, University of São Paulo Medical School, São Paulo, Brazil; ^11^Neurosurgery Service, Hospital Santa Casa, Sao Joao del Rei, Brazil; ^12^Neurosurgery Service, Santa Fe Foundation Hospital, Bogota, Colombia; ^13^Neurosurgery Service, Hospital Miguel Couto, Rio de Janeiro, Brazil

**Keywords:** neurosurgery, neurotrauma, fellowship, global neurosurgery, education

## Abstract

**Objective:** Shortage of general neurosurgery and specialized neurotrauma care in low resource settings is a critical setback in the national surgical plans of low and middle-income countries (LMIC). Neurotrauma fellowship programs typically exist in high-income countries (HIC), where surgeons who fulfill the requirements for positions regularly stay to practice. Due to this issue, neurosurgery residents and medical students from LMICs do not have regular access to this kind of specialized training and knowledge-hubs. The objective of this paper is to present the results of a recently established neurotrauma fellowship program for neurosurgeons of LMICs in the framework of global neurosurgery collaborations, including the involvement of specialized parallel education for neurosurgery residents and medical students.

**Methods:** The Global Neurotrauma Fellowship (GNTF) program was inaugurated in 2015 by a multi-institutional collaboration between a HIC and an LMIC. The course organizers designed it to be a 12-month program based on adapted neurotrauma international competencies with the academic support of the Barrow Neurological Institute at Phoenix Children's Hospital and Meditech Foundation in Colombia. Since 2018, additional support from the UK, National Institute of Health Research (NIHR) Global Health Research in Neurotrauma Project from the University of Cambridge enhanced the infrastructure of the program, adding a research component in global neurosurgery and system science.

**Results:** Eight fellows from Brazil, Venezuela, Cuba, Pakistan, and Colombia have been trained and certified *via* the fellowship program. The integration of international competencies and exposure to different systems of care in high-income and low-income environments creates a unique environment for training within a global neurosurgery framework. Additionally, 18 residents (Venezuela, Colombia, Ecuador, Peru, Cuba, Germany, Spain, and the USA), and ten medical students (the United Kingdom, USA, Australia, and Colombia) have also participated in elective rotations of neurotrauma and critical care during the time of the fellowship program, as well as in research projects as part of an established global surgery initiative.

**Conclusion:** We have shown that it is possible to establish a neurotrauma fellowship program in an LMIC based on the structure of HIC formal training programs. Adaptation of the international competencies focusing on neurotrauma care in low resource settings and maintaining international mentoring and academic support will allow the participants to return to practice in their home-based countries.

## Introduction

According to a recent analysis of global neurosurgery academic groups, more than two-thirds of the world's population has gaps in the provision of appropriate surgical and anesthetic care (1). There are an estimated 13,8 million potential neurosurgical cases every year worldwide, with more than 80% occurring in low- and middle-income countries (LMIC) (1). It has also been calculated that nearly 45% of the total neurosurgical cases are related to traumatic brain injury (TBI) care, including burr holes, craniotomies, and craniectomies (1, 2).

Unfortunately, neurosurgical care availability is limited in the most needed regions of the world. Even if neurosurgical care is available, sub-specialized care is often lacking according to global neurosurgery research audits (3–6). In general, neurotrauma surgical procedures are considered first-line macro-neurosurgical interventions according to the World Federation of Neurosurgical Societies (WFNS) classification for the level of provided neurosurgical care in different cities of the world (4).

As simple as it may seem, the success in survival and decreased disability for TBI patients is linked more with a sophisticated approach rather than the surgical procedure *per se* (7, 8). Decision-making for optimal surgical technique, selection of medical therapies, interpretation of basic and advanced neuromonitoring, and how to triage and select patients according to evidence-based outcomes in the different types of context is something that only comes with appropriate sub-specialized training in neurotrauma care (9, 10). Despite these elements, it has been recognized that formal neurotrauma fellowship programs are concentrated in HICs. Regularly, these programs are fully funded by universities or industry sponsors, and to apply, candidates need to fulfill requirements and obtain credentialing for practice under the rules of each country. As has been experienced by many, once LMIC applicants obtain credentialing for practice as sub-specialists in these countries, they seldom return to their natal countries, looking for a better way of life in high resource environments (11, 12). The objective of this paper is to present the results of a recently established model for a neurotrauma fellowship program, created for LMIC neurosurgeons within the framework of global neurosurgery collaborations.

## Materials and Methods

The Global Neurotrauma Fellowship (GNTF) program was developed in 2015 as a capacity-building effort by a multi-institutional collaboration between the Barrow Neurological Institute at Phoenix Children's Hospital in the United States and Meditech Foundation, a research center and educational non-government organization in Colombia. The original idea was established because of different discussions and clinical interactions of a mentor-mentee relationship in the framework of a capacity-building trauma grant for LMICs, sponsored by the United States (US) National Institute of Health (NIH) Fogarty International Center (D43 TW007560).

This U.S.-Colombia collaboration established the Trauma and Injury Excellence in Education on Research (TrainEER) Program, devoted to advancing the training of health care professionals in trauma research across Colombia. Through a combination of short- and long-term training, the TrainEER Program was able to provide individuals with extensive trauma and injury research training between 2007 and 2012, all while developing sustainable research partnerships with US investigators. The principal investigators were an American trauma surgeon and a Colombian trauma surgeon, Drs. Timothy Billiar and Juan C. Puyana, based at the University of Pittsburgh Medical Center in Pittsburgh (PA).

During this program, a mentor-mentee collaboration was established between Dr. David Adelson, who was the director of the Center for Injury Research and Control (CIRCL) at the University of Pittsburgh, and Dr. Andres M. Rubiano, who was a TrainEER Program fellow at the time. The collaboration was initiated during the TrainEER Program and was maintained throughout its course and beyond.

The fellowship's initial concept was established in 2012 after Dr. Adelson visited Colombia where he and Dr. Rubiano identified an opportunity to adapt the curriculum for neurotrauma fellows from the accredited Committee on Advanced Subspecialty Training (CAST) of the Society of Neurological Surgeons in the United States for implementation in low-resource environments. With private donor support from the US, MEDITECH Foundation in Colombia and Barrow Neurological Institute at Phoenix Children's Hospital in the US adopted a competency-based curriculum, ensuring trainees fulfilled requirements for basic and advanced neurotrauma care in low, medium, and high resource scenarios. A 12-month modular program (Table 1) was instituted, which included multiple clinical and surgical rotations in Colombia, Brazil, and the US.

**Table 1:** Global Neurotrauma International Fellowship Program academic sections, rotation sites, and modules.

**Program sections**	Neurotrauma general principles^*^	Research basic training^**^	General principles for global health^***^
Module 1	General Trauma Care/Clinical Skills and Surgery of Neurotrauma Care. Topics: Trauma systems, Prehospital care, Trauma resuscitation, airway management, abdominal and thoracic trauma assessment, FAST technique, orthopedic trauma assessment, imaging lecture in general trauma, pediatric and elderly trauma assessment. (Months 1-3) (Valle Salud Clinic, Cali, Colombia)	Clinical Research and Basic Science Research. Topics: best practices in clinical research, methodological approaches, basic epidemiology, basic statistical analysis, funding opportunities, ethics principles in clinical research, observational studies, clinical studies. (Months 1-3) (Meditech Foundation, Research Center, Cali, Colombia)	Leadership and Teamwork. Topics: leadership skills, phenomenological and ontological models of leadership, global health principles, humanitarian medicine, basic public health principles, teamwork. (Month 4) (Meditech Foundation, Research Center, Cali, Colombia)
Module 2	Special Procedures in Neurotrauma. Topics: Basic and advanced ICP monitoring, transcranial doppler, Infrascanner in neurotrauma, optic nerve ultrasound, neurotrauma surgical management (cranial and spinal), brain oxygenation monitoring, external ventricular drains, scalp injuries management, CSF leaks management, CNS infection management. (Months 5-6) (Valle Salud Clinic, Cali, Colombia/Addenbrooke Hospital, Cambridge, UK)	Presentation Skills at Scientific Meetings. Topics: design and development of slides and lectures, principles of health education and teaching skills. (Months 5-6) (Meditech Foundation, Research Center, Cali, Colombia)	Global Health and Global Neurosurgery. Topics: principles of global neurosurgery, neurosurgery capacity building, burden of neurosurgical diseases in LMICS, technical documents supporting global neurosurgery initiatives, task shifting and task sharing in LMICs, dealing with neurosurgical procedures in low resources environments. (Months 7-8) (UROS Clinic, Neiva, Colombia/Hospital Santa Casa Sao Joao del Rei, Brasil/Hospital Miguel Couto, Rio do Janeiro, Brasil)
Module 3	Advanced Monitoring and Neurointensive Care in Special Populations. Topics: neuromonitoring in children, EEG evaluation, evoked potentials, NIRS principles in neurointensive care, advanced imaging interpretation in neurotrauma: Special MRI sequences, angiography, tractography, biomarkers evaluation. (Months 9-10) (Barrow Neurological Institute at St Joseph and Phoenix Children Hospitals/Las Americas Clinic, Medellín, Colombia)	Skills for Scientific Writing in Biomedical Publications. Topics: general principles of narrative revies, scoping reviews, systematic reviews, meta-analysis, how to write scientific papers, reference managers, ethics in medical writing. (Months 11-12) (Meditech Foundation, Research Center, Cali, Colombia)	Neurotrauma Quality Improvement and Patient Safety. Topics: guidelines and protocol development and adherence in Neurotrauma, trauma QI methodologies, best clinical practices in neurotrauma, indicators of quality and performance in neurotrauma, morbidity and mortality panels and meetings, neurotrauma registries development and management. (Months 11-12) (Vallesalud Clinic, Cali, Colombia)

* *The neurotrauma general principles section, include a permanent rotation in surgical theater and Intensive Care Units (ICU) in different centers, from low to high level of resources for the care of neurotrauma patients*.

** *The basic research program include a mentorship program with senior academic instructors of the program, including participation in seminars, webinars and symposiums. Periodical meetings with the research group of MEDITECH and direct participation as co-investigators in active research projects in different centers are included*.

*** *The general principles for Global Health section include a mentorship program with senior academic instructors of the program, including participation in seminars, webinars, and symposiums. Periodical meetings with the research group of MEDITECH and direct participation in global surgery activities are included*.

Instructors were selected by highly experienced senior neurosurgeons and critical care physicians from these countries, all of whom were committed to an academic career in neurotrauma and neurocritical subspecialties. The clinical and surgical practices were established mainly in Cali and Neiva (Colombia), at two busy private hospitals devoted to trauma care (6 months) with complementary observational rotations for advanced neuromonitoring and advanced surgical techniques as defined by those corresponding techniques carried out in the US. Direct participation in research activities, including interaction with research methodology mentors from the US, was also part of the program.

In 2018, additional support for the continuation of the fellowship program was received from the NIHR Global Health Research in Neurotrauma Project from the University of Cambridge in the United Kingdom (UK) as part of a global neurosurgery collaboration for capacity building in neurotrauma care for LMICs. This collaboration between Colombia, the US, and the UK enhanced the infrastructure and learning opportunities of the program, adding a research component in global neurosurgery and system science. A senior expert in neurotrauma care, Prof. Peter Hutchinson, and a junior expert in clinical research, Dr. Angelos Kolias, from the University of Cambridge were added to the board of directors of the program, giving new insights into the development of the vision and mission of the fellowship moving forward.

The global neurosurgery concepts and system science were included as pivotal aspects of the future impact of this program. Within this frame, procedures can be learned in different scenarios, but the integration of public policy, global surgery, and system ecology will open a new door in knowledge and leadership projection for these fellows. The hope is that the fellows will then be prepared to create lasting changes in the health care infrastructure of their home countries equipped with leadership in knowledge and public policy skills.

Any neurosurgeon from any LMIC can apply to the fellow program, and the selection will include evaluation of the curriculum vitae and an interview with the fellowship directors. To start the process, an online application form needs to be filled including all the additional documents that are required, including curriculum vitae, personal statement, letters of recommendation, and certification of a neurological surgery program in the applicant's original country. There is no application deadline, and all the applications received during the previous year will enter the selection process for the next year. Applications are managed online at the fellowship website: https://www.globalneurotraumafellowship.com/.

## Results

Overall, eight fellows have taken part in the program. Table 2 presents the characteristics of each fellow, including their name, age range during the fellowship, home country, home institution, specialty, length of neurosurgical training, and neurosurgical experience.

**Table 2:** Characteristics of past and current fellows.

**Name**	**Country**	**Institution**	**Specialty**	**Duration of residency program in years**	**Age range**	**Time of working experience before the fellowship (in years)**
Raul Augusto Echeverri MD	Venezuela	Institute of Neurology and Neurosurgery, La Havana, Cuba	Neurosurgery	5	30-40	5
Ahsan Ali Khan MD	Pakistan	Southeast University, Nanjing, China	Neurosurgery	4	20-30	1
Santiago Morales MD	Colombia	El Bosque University, Colombia	Neurosurgery	5	40-50	15
Diana Marcela Sánchez Parra MD	Colombia	Centro Internacional de Restauracion Neurologica, La Havana, Cuba	Neurosurgery	5	30-40	1
Jorge Luiz Da Rocha Paranhos MD	Brazil	Brazilian Medical Association	Neurosurgery/Critical Care	5	60-70	26
José Nel Carreño MD	Colombia	Juan N. Corpas School of Medicine	Neurosurgery/Critical Care	5	40-50	23
Wellingson Silva Paiva MD, PhD	Brazil	University of São Paulo	Neurosurgery	5	40-50	11
Robson Luis Oliveira de Amorim MD, PhD	Brazil	University of São Paulo	Neurosurgery	5	40-50	11

All fellows were funded, with a salary stipend, in addition to the provision for housing, food, and transportation while on international rotations. Additional financial support was provided for research training. This included funding for data collection, management, analysis, and support for academic writing.

### Clinical Exposure

**Figure 1:**
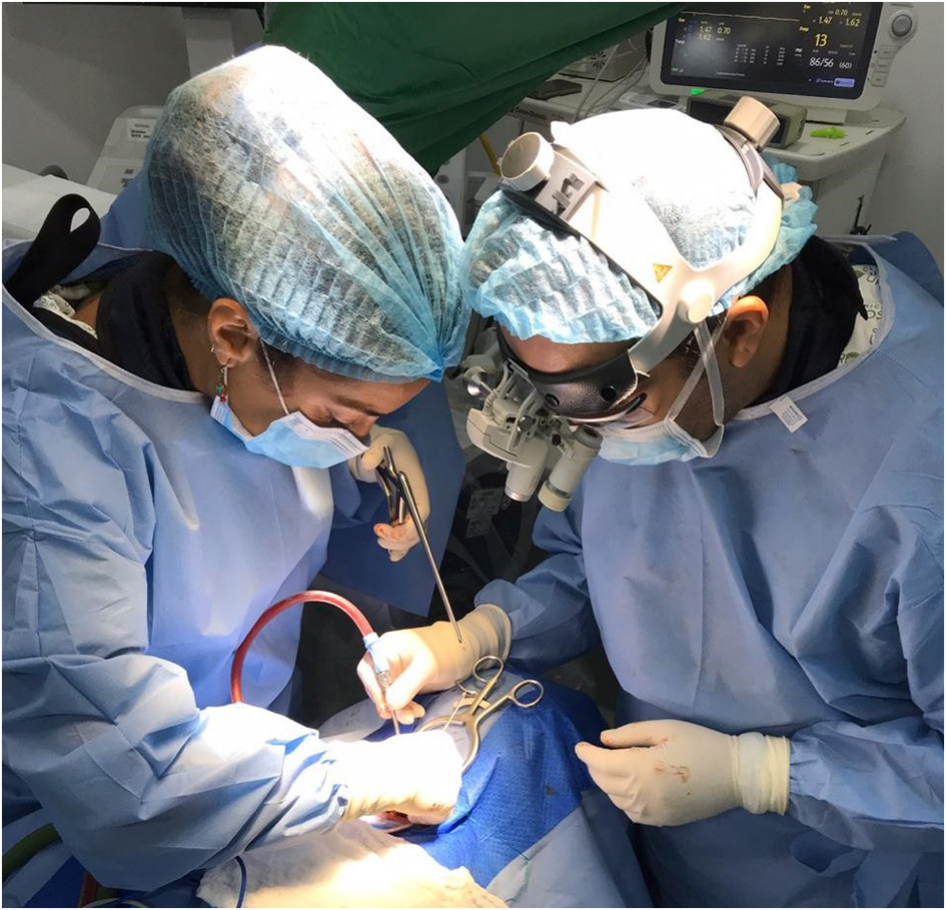
Dr. Laura Pastor, visiting neurosurgical resident from Spain, performing a spinal cord injury surgery with a mentor of the fellowship program in Colombia, Dr. Alvaro Soto.

All fellows were exposed to basic and advanced training at several institutions with neurosurgery and critical care capabilities in Colombia (Vallesalud Clinic, Cali/UROS Clinic, Neiva/Las Americas Clinic, Medellin), in addition to lower-level facilities with solely base-level neurosurgical capabilities (Figure 1). Some of these rotations include the trauma centers in the cities of Bogota, Neiva, Cali, and Medellin. Fellows have also rotated in busy trauma centers in Brazil (Hospital Miguel Couto, Rio do Janeiro/Hospital Santa Casa, Sao Joao del Rei/Hospital Das Casas, São Paulo), and the US and UK (Barrow Neurological Institute at Phoenix Children Hospital and St Joseph Hospital in Phoenix/Addenbrookes Hospital, Cambridge, UK).

### Continuous Medical Education

Fellows have taken part in clinical research training programs and continuing medical education (CME) programs such as the advanced trauma life support (ATLS) course of the American College of Surgeons, the advanced cardiac life support (ACLS) course of the American Heart Association, the Fundamental Critical Care Course (FCCS) course of the American Critical Care Society and the Trauma Ultrasound Training Program (USET) of the Pan-American Trauma Society.

### Research

Fellows have contributed significantly to the global neurosurgery academic community, presenting their research at international conferences such as the International Conference for Recent Advances in Neurotraumatology (ICRAN) and publishing in highly reputable journals like NEUROSURGERY, Frontiers in Neurology, and World Neurosurgery (13–16). They perform most of the research activities while based in Colombia in different centers in a broad level of resource availability. Everything to apply similar principles when they return to their home countries. Quality improvement research activities are under planning to evaluate the impact of the fellow's training after finishing the program.

### Post-fellowship

**Figure 2:**
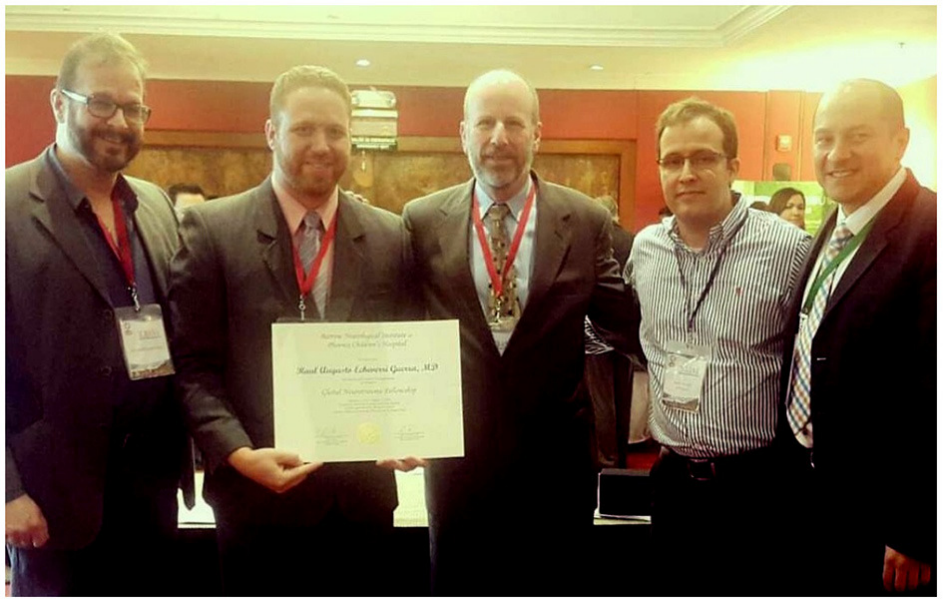
Graduation ceremony of Dr. Raul Echeverri (Venezuela), who created an award for Venezuelan neurosurgical residents who traveled to Colombia for additional neurotrauma training within the fellowship program. From left to right, Dr. Ruy Monteiro (Brazil), Dr. Echeverri (Venezuela), Dr. David Adelson (US), Dr. Alvaro Soto (Colombia), and Dr. Andres M. Rubiano (Colombia).

The fellows that have completed the program have gone on to create lasting change in their communities. One fellow created a program of sponsored rotations in neurotrauma and critical care for Venezuelan residents who return to their country to improve neurotrauma care practices in different regions throughout the country (Figure 2). We consider these actions as a means of measuring the program's overall impact. Other fellows have been strong advocates for the improvement of neurotrauma health-system strengthening in their home institutions, most notably in Brazil, where one of our fellows has been actively leading neurotrauma care research and systems improvements activities in the Amazonian region.

## Discussion

Neurotrauma care in low-resource settings faces many challenges. This includes a lack of availability of subspecialty care for neurosurgeons, neuro-intensivist, and neuroscience nursing. There is a lack of staff, disparities in distribution, and access to adequate technologies for diagnosis and management, including the basics of CT scanning and ICU care. Furthermore, there is a lack of financial resources for the long-term care of patients following all levels of neurotrauma (17–19).

A large portion of this disparity of care could be mitigated by an increase in neurotrauma and neurocritical care staff. However, in HICs, there has been an overall lack of interest in these subspecialties. This general lack of interest from HIC's has been transferred to LMIC's despite neurotrauma being identified as one of the neurologic and neurosurgical conditions with the most substantial patient burden in LMIC's. This brings with it the most significant opportunities for advancement care. Nevertheless, young neurosurgeons seem to have more motivation for highly technological driven specialties like functional neurosurgery, endovascular, cerebrovascular, and skull-based neurosurgery (20–22). These, along with other highly reimbursing specialties like spine or tumor neurosurgery are incredibly lucrative and thus, highly sought after. The subspecialties tend to bring with them a more comfortable lifestyle, including less of a load of emergency procedures during nights and weekends and a more significant economic return for those pursuing private and elective practice.

Neurotrauma care has been traditionally offered in public and charity institutions in all countries. Many of these institutions regularly face economic and administrative problems when compared to their private hospital, practice, and institutional counterparts (23–25). In this way, neurotrauma care may be perceived as a second-class subspecialty, reserved for trainees in entry-level neurosurgery. Additionally, there is often a lack of primary focus for neurotrauma in neurocritical care at national and international meetings. This further extends the misperception that neurotrauma is a second-class subspecialty. This has led to separate organizations and specialties developing a framework to fill this gap, i.e., neurocritical care or trauma care general societies without the involvement of neurosurgery (26–28).

**Figure 3:**
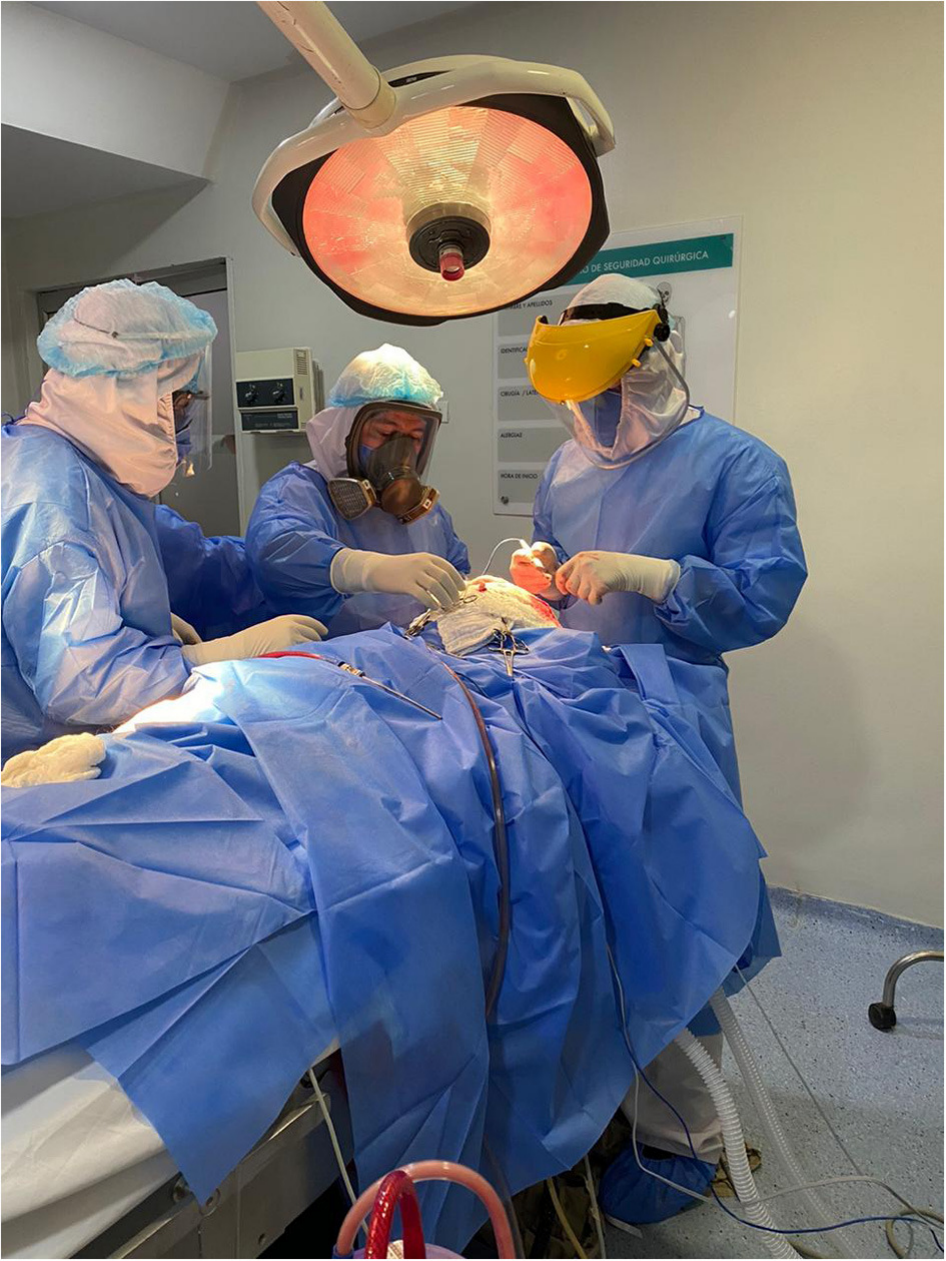
Trauma surgery during COVID-19 pandemic. Dr. Santiago Morales, neurotrauma fellow (Colombia), and Dr. Andres M. Rubiano, fellow instructor (Colombia) performing an emergency cranial decompression on a patient with severe TBI.

With the disproportionately high burden of neurotrauma disease in LMIC's, neurotrauma is a highly technical and complex subspecialty in these geographic areas where it is most needed (Figure 3). In the absence of formal neurotrauma subspecialty training programs in these areas, it is easy for this view to grow. It is not uncommon to see formal fellowship training programs in endovascular or spine neurosurgery in LMICs, with an absence of formal neurotrauma fellows (29, 30). In these countries, the national burden of neurotrauma has an incidence of 800-939 cases for every 100,000 people. This, in contrast to the incidence of aneurysms and cervical stenosis in these countries as 3.4-6.9 cases for every 100,000 and 2.3-3.4 cases for every 100,000, respectively (2, 31, 32).

The question that arises is whether the available short-term rotations or short-term programs or CME activities would provide enough expertise to fill this gap. A review of the existing curriculums for neurotrauma and neurocritical care does not fulfill the requirements of an appropriate training program that will allow fellows to achieve mastery in the triage/assessment of the neurotrauma patient, the decision making for medical and surgical care, the integration of care pathways, and the surgical skills in innovation for the future growth and improvement of inpatient care (33, 34). Many severe TBI patients are not appropriately managed because they are “too sick,” or moderate TBI patients are not aggressively managed because they are considered “not sick enough.” When a neurotrauma patient dies, the “fulfilled prophecy” is accomplished (35).

With a new area of global neurosurgery forged by discoveries in system science research, stakeholders can see more clearly the many moving parts and processes that both strengthen and weaken health systems and thus neurotrauma care. System science understands that neurotrauma care, like any other disease, is a function of the multitude of factors that go into providing optimal care. This includes healthcare personnel, technology, equipment, infrastructure, health guidelines, and processes, in addition to educational programming. The interaction of these components is what determines optimal vs. suboptimal care.

Of these factors, educational programming has both an immediate and a future-looking approach as fellows are involved in care in real-time and are the future not only of the field itself but also as the next generation of educators. A formal education program will allow fellows to learn the complexities of care, but also the moving parts revealed by system science research. When solely focusing on general neurosurgical aspects of care, essential elements for the proper care of neurotrauma patients can easily be overlooked (36, 37). Aspects like timely access to early surgery, appropriate interaction with prehospital and emergency providers, teamwork with trauma surgeons or other critical care physicians are essential aspects of the job, and thus education must not overlook these areas (38).

The fellowship program that we have developed exposes neurosurgeons from LMICs to a full range of contexts where they can become engaged as part of a broader community of peers within the same subspecialty and can share in the challenges and successes that come with practicing with different levels of resources and perspectives. Working in this environment allows them to participate in the global community of neurotrauma and neurocritical care providers. They participate in the same clinical and academic activities, and partner and collaborate on research projects, focusing on creating a difference in their home regions. Since the inception of the program, the mentoring process has fostered that transformational experience for LMIC neurosurgeons. Our next step is to add new competencies from the human aspect, including leadership training and communication skills that are critical when facing difficult situations like managing severe TBI patients in low resources settings.

Multinational and multi-institutional collaborations like the Global Health Research Group in Neurotrauma are creating new possibilities to enhance these types of programs to create the next generation of neurotrauma care leaders in LMICs. Initiatives include funding fellows in LMICs to undertake specific research projects and to collect data for extensive observational studies (39). The current list of fellows and projects from the NIHR program covers a wide range of collaborations in and with LMICs (Table 3). Other examples of trainee engagement include participation in the World Health Assembly of United Nations, organized by the WHO and held annually in Geneva, to assist in raising the profile of neurological and surgical disorders with health ministers and ministries globally (40, 41). Many of the different aspects of the program can be measured by generating indicators of impact for this program, including the number of new collaborations created by fellows in their home countries, number, and quality of publications addressing global surgery and public health aspects from the neurotrauma perspective, or new funding obtained for support of research activities in their home countries.

**Table 3:** National Institutes of Health Research—Global Health Research Group in Neurotrauma. University of Cambridge.

**List of supported fellows 2018/2019, NIHR Global Health Research Group**			
**on Neurotrauma, University of Cambridge**			
**Country**	**Gender (** ***n*** **)**	**Age range**	**Institution**
Brazil	Male (1)	(20-40)	University of São Paulo
Colombia	Male (2) Female (1)	(20-40)	Meditech Foundation
Ethiopia	Female (2)	(20-40)	Addis Ababa University &Tikur Anbessa Hospital
India	Male (3)	(20-40)	National Institute of Mental Health & Neurosciences Christian Medical College
Indonesia	Male (1)	(20-40)	Soetomo General Hospital Airlangga University
Malaysia	Male (2)	(20-40)	University of Malaya Medical Centre
Myanmar	Male (1) Female (1)	(20-40)	Yangon, University of Medicine 1
Pakistan	Male (1)	(20-40)	North West General Hospital and Research Center
South Africa	Male (1)	(20-40)	Red Cross Children's Hospital and University of Cape Town
Tanzania	Male (1)	(20-40)	Muhimbili Orthopaedic Institute
United Kingdom	Male (3)	(20-40)	Cambridge University Hospitals NHS Trust
Zambia	Male (1)	(20-40)	University Teaching Hospital

*List of sponsored GHRG Research Fellows in Neurotrauma Clinical Research (2018-2019)*.

The Fellowship Program has not been affected by the Covid pandemic, as most trauma cases have continuing arriving at most hospitals. Telemedicine initiatives have been involved to accomplish a better evaluation of patients under risk circumstances. There are recent publications produced by the fellowship group related to exploring what are the ideal conditions to keep safety measures during emergency neurotrauma surgery (42, 43).

## Conclusion

It is possible to establish a neurotrauma fellowship program in an LMIC based on the established criteria for neurotrauma training in HIC programs. Adaptation of the international competencies focusing on neurotrauma care in low resource settings and maintaining international mentoring and academic support enhances the likelihood that the neurotrauma-trained fellow will return to clinical and academic practice in their home countries. Ongoing collaborative global community support will further enhance the growth of the advanced level of care in LMICs.

## Data Availability Statement

The original contributions presented in the study are included in the article/supplementary material, further inquiries can be directed to the corresponding author/s.

## Author Contributions

AR, DG, PA, RE, AK, SM, AAK, and PH contributed to the design and implementation of the research, to the analysis of the results, and to the writing of the manuscript. All authors contributed to the article and approved the submitted version.

## Conflict of Interest

The authors declare that the research was conducted in the absence of any commercial or financial relationships that could be construed as a potential conflict of interest.

## Publisher's Note

All claims expressed in this article are solely those of the authors and do not necessarily represent those of their affiliated organizations, or those of the publisher, the editors and the reviewers. Any product that may be evaluated in this article, or claim that may be made by its manufacturer, is not guaranteed or endorsed by the publisher.
